# On Rationality of Kneading Determinants

**DOI:** 10.1155/2014/504160

**Published:** 2014-02-18

**Authors:** Sheng Chen, Chao Xia

**Affiliations:** Department of Mathematics, Harbin Institute of Technology, Harbin 150001, China

## Abstract

In this section, *R* denotes a ring with identity *I*. We study conditions under which *I* − *Aλ* and *I* − *Bλ* are left coprime or right coprime, where *A*, *B* ∈ *R*. As applications, we get sufficient conditions under which the Kneading determinant of a finite rank pair of operators on an infinite dimensional space is rational.

## 1. Introduction

If *A* is a *k* × *k* matrix with rational coefficients, one has the well-known identity between formal power series:

(1) $$\begin{array}{c} \exp\underset{n\geq 1}{\sum }-\frac{\mathrm{tr}\left(A^{n}\right)}{n}z^{n}=\det\left(I-zA\right), \end{array}$$

where tr⁡(*A* ^*n*^) denotes the trace of matrix *A* ^*n*^ (matrix *A* raised to the *n*th power) and *I* denotes the *k* × *k* identity matrix. This identity plays a significant role in the discussion of an important problem in dynamical systems theory. For more details see [1, 2].

We denote by *ℋ* the infinite dimension vector space over *ℚ*; the space of linear forms on *ℋ* will be denoted, as usual, by ℋ*, and the space of all linear endomorphisms on *ℋ* will be denoted by *L*(*ℋ*). If *ψ* ∈ *L*(*ℋ*) and *n* is a nonnegative integer, the *n*th iterate *ψ* ^*n*^ is defined recursively by *ψ* ^0^ = Id_*ℋ*_ ∈ *L*(*ℋ*), *ψ* ^*n*^ = *ψ*∘*ψ* ^*n*−1^, for *n* ≥ 1.

The subspace of *L*(*ℋ*) whose elements are the linear endomorphism on *ℋ* with finite rank will be denoted by *L*_FR_(*ℋ*).

Let *q* be a positive integer; we use the symbol $\bar{h}$ to denote an element of *ℋ* ^*q*^ = *ℋ* × *ℋ* × *ℋ* × ⋯×*ℋ* (*q* times) and the symbol $\bar{\alpha }$ to denote an element of *ℋ* ^∗*q*^; that is

(2) $$\begin{array}{c} \bar{h}=\left(h_{1},\ldots ,h_{q}\right),\;\;\bar{\alpha }=\left(\alpha_{1},\ldots ,\alpha_{q}\right). \end{array}$$

Given $\bar{h}\in {ℋ}^{q}$ and $\bar{\alpha }\in {ℋ}^{*q}$, we define the finite rank endomorphism $\bar{\alpha }⊗\bar{h}\in L_{\text{FR}}(ℋ)$ and the matrix $M(\bar{\alpha },\bar{h})\in {\mathbb{Q}}^{q\times q}$ by setting

(3) $$\begin{array}{c} \bar{\alpha }⊗\bar{h}=\overset{q}{\underset{p=1}{\sum }}\alpha_{p}⊗h_{p}, \end{array}$$

with the usual notation

(4) $$\begin{array}{c} \alpha \in {ℋ}^{*},h\in ℋ:\left(\alpha ⊗h\right)\left(x\right)=\alpha \left(x\right)u,\;x\in ℋ,\\ M\left(\bar{\alpha };\bar{h}\right)=\left(\begin{array}{ccc} \alpha_{1}\left(h_{1}\right) & \cdots & \alpha_{1}\left(h_{q}\right)\\ \vdots & \ddots & \vdots \\ \alpha_{q}\left(u_{1}\right) & \cdots & \alpha_{q}\left(h_{q}\right) \end{array}\right). \end{array}$$

**Definition 1 (see [3])**

A pair (*φ*, *ψ*) ∈ *L*(*ℋ*) × *L*(*ℋ*) is said to have finite rank if *ψ* − *φ* ∈ *L*_FR_(*ℋ*).

Notice that if a pair (*φ*, *ψ*) has finite rank, then the pair (*φ* ^*n*^, *ψ* ^*n*^) also has finite rank for all *n* ≥ 1, and therefore the trace of *φ* ^*n*^ − *ψ* ^*n*^ is defined.

**Definition 2 (see [3])**

For any pair (*φ*, *ψ*) ∈ *L*(*ℋ*) × *L*(*ℋ*) with finite rank, we define the Kneading determinant of (*φ*, *ψ*) as the following invertible element of *ℚ*[[*z*]]:

(5) $$\begin{array}{c} \Delta_{\left(\varphi ,\psi \right)}=\exp\underset{n\geq 1}{\sum }\frac{\mathrm{tr}\left(\varphi^{n}-\psi^{n}\right)}{n}z^{n}. \end{array}$$

**Remark 3**

Kneading determinant was first studied by Milnor and Thurston in [4].

Let *ℚ*[[*z*]]^*q*×*q*^ be the ring of the *q* × *q* matrices whose entries lie in *ℚ*[[*z*]]. If *φ* ∈ *L*(*ℋ*), $\bar{u}\in {ℋ}^{q}$, and $\bar{\alpha }\in {ℋ}^{*q}$, we define the matrix $M_{\varphi }(\bar{\alpha };\bar{u})\in \mathbb{Q}{\left[[z]\right]}^{q\times q}$ by

(6) $$\begin{array}{c} M_{\varphi }\left(\bar{\alpha };\bar{u}\right)=\left(\begin{array}{ccc} \underset{n\geq 0}{\sum }\alpha_{1}\varphi^{n}\left(u_{1}\right)z^{n} & \cdots & \underset{n\geq 0}{\sum }\alpha_{1}\varphi^{n}\left(u_{q}\right)z^{n}\\ \vdots & \ddots & \vdots \\ \underset{n\geq 0}{\sum }\alpha_{q}\varphi^{n}\left(u_{1}\right)z^{n} & \cdots & \underset{n\geq 0}{\sum }\alpha_{q}\varphi^{n}\left(u_{q}\right)z^{n} \end{array}\right). \end{array}$$

**Lemma 4 (see [3])**

Let (*φ*, *ψ*) ∈ *L*(*ℋ*) × *L*(*ℋ*), $\bar{u}\in {ℋ}^{q}$, and $\bar{\alpha }\in {ℋ}^{*q}$ such that $\psi -\varphi =\bar{\alpha }⊗\bar{u}$. Denote by **I** the *q* × *q* identity matrix. Then,

(7) $$\begin{array}{c} \Delta_{\left(\varphi ,\psi \right)}=\det\left(I-zM_{\varphi }\left(\bar{\alpha };\bar{u}\right)\right) \end{array}$$

holds in *ℚ*[[*z*]].

In general, any power series can be the Kneading determinant of some pair (*φ*, *ψ*) with finite rank (see [3]). So it is interesting to study conditions under which the Kneading determinant is a rational power series.

The following is the main result of this paper.

**Theorem 5**

If *I* − *φλ* and *I* − *ψλ* are left coprime or right coprime, and *h* = *ψ* − *φ* with finite rank, then Δ_(*φ*,*ψ*)_ is a rational function.

## 2. Coprimeness of *I* − *Aλ* and *I* − *Bλ*

In this section, *R* denotes a ring with identity *I*. We discuss conditions under which *I* − *Aλ* and *I* − *Bλ* are left coprime or right coprime, where *A*, *B* ∈ *R*.

We say *I* − *Aλ* and *I* − *Bλ* are left coprime if there exist polynomials *X*(*λ*), *Y*(*λ*) ∈ *R*[*λ*] such that

(8) $$\begin{array}{c} X\left(\lambda \right)\left(I-A\lambda \right)+Y\left(\lambda \right)\left(I-B\lambda \right)=I. \end{array}$$

**Proposition 6**

*I* − *Aλ* and *I* − *Bλ* are left coprime if and only if there exist *X*_0_, *X*_1_,…, *X*_*m*_ ∈ *R* such that

(9) $$\begin{array}{c} X_{m}H+X_{m-1}HB+X_{m-2}HB^{2}+\cdots +X_{0}HB^{m}+B^{m+1}=0, \end{array}$$

for *H* = *A* − *B*.

**Proof**

If *I* − *Aλ* and *I* − *Bλ* are left coprime, there exist *X*(*λ*) = *X*_0_ + *X*_1_ *λ* + ⋯+*X*_*m*_ *λ* ^*m*^ and *Y*(*λ*) = *Y*_0_ + *Y*_1_ *λ* + ⋯+*Y*_*s*_ *λ* ^*s*^ such that

(10) $$\begin{array}{c} X\left(\lambda \right)\left(I-A\lambda \right)+Y\left(\lambda \right)\left(I-B\lambda \right)=I. \end{array}$$

We can assume that *s* equals *m*. And we have

(11) $$\begin{array}{c} X_{0}+Y_{0}=I,\\ X_{i}A+Y_{i}B-X_{i+1}-Y_{i+1}=0,\;i=1,2,\ldots ,m-1,\\ X_{m}A+Y_{m}B=0. \end{array}$$

So,

(12) $$\begin{array}{c} Y_{0}=I-X_{0}. \end{array}$$

If we write *H* = *A* − *B*, then,

(13) $$\begin{array}{c} Y_{1}=-X_{1}+X_{0}H+B,\\ Y_{2}=-X_{2}+X_{1}H+X_{0}HB+B^{2},\\ \vdots \\ Y_{m}=-X_{m}+X_{m-1}H+\cdots +X_{0}HB^{m-1}+B^{m}. \end{array}$$

So,

(14) $$\begin{array}{c} X_{m}H+X_{m-1}HB+X_{m-2}HB^{2}+\cdots +X_{0}HB^{m}+B^{m+1}=0. \end{array}$$

Conversely, if there exist *X*_0_, *X*_1_,…, *X*_*m*_ ∈ *R* such that

(15) $$\begin{array}{c} X_{m}H+X_{m-1}HB+X_{m-2}HB^{2}+\cdots +X_{0}HB^{m}+B^{m+1}=0, \end{array}$$

let

(16) $$\begin{array}{c} X\left(\lambda \right)=X_{0}+X_{1}\lambda +\cdots +X_{m}\lambda^{m},\\ Y_{0}=I-X_{0},\\ Y_{1}=-X_{1}+X_{0}H+B,\\ Y_{2}=-X_{2}+X_{1}H+X_{0}HB+B^{2},\\ \vdots \\ Y_{m}=-X_{m}+X_{m-1}H+\cdots +X_{0}HB^{m-1}+B^{m}, \end{array}$$

and let

(17) $$\begin{array}{c} Y\left(\lambda \right)=Y_{0}+Y_{1}\lambda +\cdots +Y_{m}\lambda^{m}. \end{array}$$

Then,

(18) $$\begin{array}{c} X\left(\lambda \right)\left(I-A\lambda \right)+Y\left(\lambda \right)\left(I-B\lambda \right)=I. \end{array}$$

Now we give the definition of right coprime. We say *I* − *Aλ* and *I* − *Bλ* are right coprime if there exist polynomials *X*(*λ*), *Y*(*λ*) ∈ *R*[*λ*] such that

(19) $$\begin{array}{c} \left(I-A\lambda \right)X\left(\lambda \right)+\left(I-B\lambda \right)Y\left(\lambda \right)=I. \end{array}$$

**Proposition 7**

*I* − *Aλ* and *I* − *Bλ* are right coprime if and only if there exist *X*_0_, *X*_1_,…, *X*_*s*_ ∈ *R* such that

(20) $$\begin{array}{c} HX_{s}+BHX_{s-1}+B^{2}HX_{s-2}+\cdots +B^{s}HX_{0}+B^{s+1}=0, \end{array}$$

for *H* = *A* − *B*.

**Proof**

It is similar to the proof of Proposition 6.

## 3. Proof of the Main Theorem

In this section, *φ*, *ψ* ∈ *L*(*ℋ*), where *ℋ* is an infinite dimensional vector space over *ℚ*, and we denote by *L*(*ℋ*) the ring of linear transforms on *ℋ*.

**Lemma 8**

Suppose that *I* − *φλ* and *I* − *ψλ* are left coprime or right coprime, *h* = *ψ* − *φ* is of finite rank, and *W* = Im (*h*). Then there exists a finite dimensional space $\tilde{W}$ containing *W* such that $(\varphi^{k}-\psi^{k})(ℋ)\subset \tilde{W}$ with *k* = 1,2,….

**Proof**

First we assume that if *I* − *φλ* and *I* − *ψλ* are right coprime, then by Proposition 7 we have *X*_0_, *X*_1_,…, *X*_*s*_ ∈ *L*(*ℋ*) such that

(21) $$\begin{array}{c} hX_{m}+\varphi hX_{m-1}+\varphi^{2}hX_{m-2}+\cdots +\varphi^{m}hX_{0}+\varphi^{m+1}=0. \end{array}$$

Write $\tilde{W}=W+\varphi W+\varphi^{2}W+\cdots +\varphi^{m}W$; $\tilde{W}$ is an invariant subspace of *ℋ* under *h*. It is not very hard to check that $\varphi (\tilde{W})=\varphi^{m+1}W+\varphi W+\varphi^{2}W+\cdots +\varphi^{m}W\subset \tilde{W}$. So $\tilde{W}$ is invariant under the operator *φ*.

We use induction to prove the conclusion.

If *k* = 1, then (*φ* ^*k*^ − *ψ* ^*k*^)(*ℋ*) = *W*.

Suppose that for *k* ≤ *l* we have (*φ* ^*k*^ − *ψ* ^*k*^)(*ℋ*) ⊂ *W* + *φW* + ⋯+*φ* ^*m*^ *W*. Then for *k* = *l* + 1, we have $(\varphi^{k}-\psi^{k})(ℋ)=\varphi (\varphi^{l}-\psi^{l})(ℋ)-h\psi^{l}(ℋ)\subset \varphi (\tilde{W})+W\subset \tilde{W}$. We get the conclusion in this case.

Now we assume that *I* − *φλ* and *I* − *ψλ* are left coprime; then there exist *s* ∈ *ℤ* and *x*_0_, *x*_1_,…, *x*_*s*_ ∈ *L*(*ℋ*) such that

(22) $$\begin{array}{c} x_{s}h+x_{s-1}h\varphi +x_{s-2}h\varphi^{2}+\cdots +x_{0}h\varphi^{s}+\varphi^{s+1}=0, \end{array}$$

holds. Take

(23) $$\begin{array}{c} \hat{W}=W+x_{0}W+\cdots +x_{s}W,\\ \tilde{W}=\hat{W}+\varphi \hat{W}+\varphi^{2}\hat{W}+\cdots +\varphi^{s}\hat{W}. \end{array}$$

Notice when *l* ≥ *s* + 1, *φ* ^*l*^(*ℋ*) ⊂ *x*_0_ *W* + *x*_1_ *W* + ⋯+*x*_*s*_ *W*.

We use induction to prove the conclusion.

If *k* = 1, then (*φ* ^*k*^ − *ψ* ^*k*^)(*ℋ*) = *W*.

Suppose that for *k* ≤ *l* we have (*φ* ^*k*^ − *ψ* ^*k*^)(*ℋ*) ⊂ *W* + *φW* + ⋯+*φ* ^*m*^ *W*. Then for *k* = *l* + 1, we have $(\varphi^{k}-\psi^{k})(ℋ)=\varphi^{l}h(ℋ)-(\varphi^{l}-\psi^{l})\psi (ℋ)\subset \varphi^{l}(W)+\tilde{W}\subset \tilde{W}$.

The proof is complete.

Now we will give the proof of Theorem 5.

**Proof**

If *I* − *φλ* and *I* − *ψλ* are coprime, by Lemma 8, there is a finite dimensional space $\tilde{W}$ such that

(24) $$\begin{array}{c} \left(\psi^{k}-\varphi^{k}\right)\left(ℋ\right)\subset \tilde{W}, \end{array}$$

and we have $\varphi (\tilde{W})\subset \tilde{W}$. Define

(25) $$\begin{array}{c} \tilde{\varphi }={\text{Pr}(\varphi )|}_{\tilde{W}}:\tilde{W}⟶\tilde{W}. \end{array}$$

Suppose *q* = rank⁡(*φ* − *ψ*); we denote by *I*_*q*_ the identity operator. Then $p(z)=\det(I_{q}-z\tilde{\varphi })\in \mathbb{Q}[z]$. For any *i*, *j* ∈ {1,2,…, *q*}, we have ∑_*n*≥0_ *α*_*i*_ *φ* ^*n*^(*u*_*j*_)*z* ^*n*^ = *f*_*ij*_(*z*)/*p*(*z*) for some *f*_*ij*_(*z*) ∈ *ℚ*[*z*]. For more details see [5]. So by Lemma 4, $\det(I-zM_{\varphi }(\bar{\alpha };\bar{u}))$ is rational; we get the conclusion.

**Example 9**

Suppose *ℋ* is the ring of countable infinite matrix with finite nonzero entries in each column.

Let

(26) $$\begin{array}{c} \varphi =\left(\begin{array}{cccc} \alpha_{1} & \alpha_{2} & \alpha_{3} & \cdots \\ A & 0 & 0 & \cdots \\ 0 & A & 0 & \cdots \\ \vdots & \vdots & \ddots & \ddots \end{array}\right),\;\;h=\left(\begin{array}{cccc} \alpha_{1} & \alpha_{2} & \alpha_{3} & \cdots \\ 0 & 0 & 0 & \cdots \\ 0 & 0 & 0 & \cdots \\ \vdots & \vdots & \ddots & \ddots \end{array}\right),\\ \psi =\varphi +h, \end{array}$$

where $A=\left(\begin{array}{ccc} 0 & 1 & 0\\ 0 & 0 & 1\\ 0 & 0 & 0 \end{array}\right)$ and *α*_1_, *α*_2_, *α*_3_,… are three-dimensional row vectors. We see that (*φ* − *h*)^3^ = 0. So *I* − *ψλ* and *I* − *φλ* are left coprime. It is easy to check that

(27) $$\begin{array}{c} \Delta_{\left(\varphi ,\psi \right)}=\frac{1-2z}{1-z}. \end{array}$$
